# Elimination of *Schistosoma japonicum* Transmission in China: A Case of Schistosomiasis Control in the Severe Epidemic Area of Anhui Province

**DOI:** 10.3390/ijerph16010138

**Published:** 2019-01-07

**Authors:** Linhan Li, Yibiao Zhou, Tianping Wang, Shiqing Zhang, Gengxin Chen, Genming Zhao, Na He, Zhijie Zhang, Dongjian Yang, Ya Yang, Yu Yang, Hongchang Yuan, Yue Chen, Qingwu Jiang

**Affiliations:** 1 Department of Epidemiology, School of Public Health, Fudan University, 130 Dong’an Road, Shanghai 200032, China; 16111020005@fudan.edu.cn (L.L.); ybzhou@fudan.edu.cn (Y.Z.); gmzhao@shmu.edu.cn (G.Z.); nhe@shmu.edu.cn (N.H.); zhj_zhang@fudan.edu.cn (Z.Z.); 17211020111@fudan.edu.cn (D.Y.); yayang16@fudan.edu.cn (Y.Y.); 16211020071@fudan.edu.cn (Y.Y.); hcyfduepi@163.com (H.Y.); 2 Key Laboratory of Public Health Safety, Ministry of Education, 130 Dong’an Road, Shanghai 200032, China; 3 Center of Tropical Disease Research, 130 Dong’an Road, Shanghai 200032, China; 4 Institute for the Prevention and Control of Schistosomiasis in Anhui Province, 337 Wuhu Road, Hefei 230061, China; wangtianping@hotmail.com (T.W.); zhangdj1994@aliyun.com (S.Z.); 5 Guichi Antischistosomiasis Station, West Qiupu Road, Chizhou 247100, China; cgx5611@163.com; 6 School of Epidemiology and Public Health, University of Ottawa, 600 Peter Morand, Ottawa, ON K1G 5Z3, Canada; Yue.Chen@uottawa.ca

**Keywords:** evaluation, method, policy, strategy, social, trend

## Abstract

Over the several decades, China has been incessantly optimizing control strategies in response to the varying epidemic situations of schistosomiasis. We evaluated continuously the changing prevalence under different control strategies of two villages, Sanlian and Guifan, in China through five phases lasting 37 years. We tested residents, calculated prevalence and discussed change causes. We found the prevalence in Sanlian did not differ significant from that of Guifan (*p* = 0.18) in 1981, but decreased to 2.66%, much lower than Guifan’s 11.25%, in 1984 (*p* = 0). Besides, prevalence in Guifan increased to 21.25% in 1987, while in Sanlian it rose to 20.78% until 1989. Those data confirmed that praziquantel combined with snail control could better reduce the prevalence. From 1992 to 1994, the prevalence in the two villages displayed downtrends, which showed the World Bank Loan Project worked. From 1995 to 2004, repeated oscillations with no obvious change trend was seen. Since 2005, the prevalence in both villages has shown a significant downtrend (*p* < 0.05), which suggests the integrated strategy is effective. We considered the control strategies were implemented suitably in the study area under changing social circumstances. Adjusting the strategy in consideration of social transformations is necessary and vital. The experience may be useful for policy making of other epidemic areas with an analogous situation.

## 1. Introduction

The archeological discovery of Schistosoma eggs in a corpse in the Changsha Mawangdui Han tomb in Hunan Province revealed that schistosomiasis has been prevalent in China for over 2000 years. Not until the American physician Logan first reported the clinical diagnosis of schistosomiasis in China [1], did Chinese people begin to consider schistosomiasis as a disease. *Schistosoma japonicum*, one of the five species of schistosome, is widely distributed in China, Indonesia, and The Philippines [2]. As it needs to go through several growth stages in water, the transmission and infection of humans is closely related to specific geographic conditions, which causes difficulties in prevalence control [3]. Schistosomiasis brings a heavy burden to China’s population health, social stability, and economic development.

After the establishment of the People’s Republic of China in 1949, the central government organized and delegated expert teams to conduct a national survey that found 12 provinces and 351 counties where schistosomiasis prevailed [4] with some 10.5–11.8 million people infected. Zedong Mao, the Chinese president back then, with immense political influence, wrote a famous poem ”Farewell to the god of plague” and launched a massive country-wide movement to eliminate schistosomiasis [5]. With medical treatment, environmental modification, and molluscicide application, by 1980, four out of the 12 provinces and two thirds of the affected counties successfully repressed the prevalence of schistosomiasis [6]. As for the remaining eight provinces, epidemic areas were mostly distributed in marshlands and mountainous areas with complicated environmental factors that hindered control.

In order to address this predicament and search for a new and effective schistosomiasis control method, Warren and Su conducted a study in Guichi district, Anhui Province, one of the ten historically most severely affected areas in China [7] and confirmed praziquantel therapy could effectively reduce the schistosomiasis prevalence in humans. When combined with snail control in epidemic areas, the prevalence decreased even more significantly. In this study, we performed a 37-years longitudinal study from 1981 to 2017 on the basis of the study conducted by Warren and Su to understand the long-term effects of schistosomiasis transmission control strategies. 

## 2. Materials and Methods

### 2.1. Study Area

**Figure 1:**
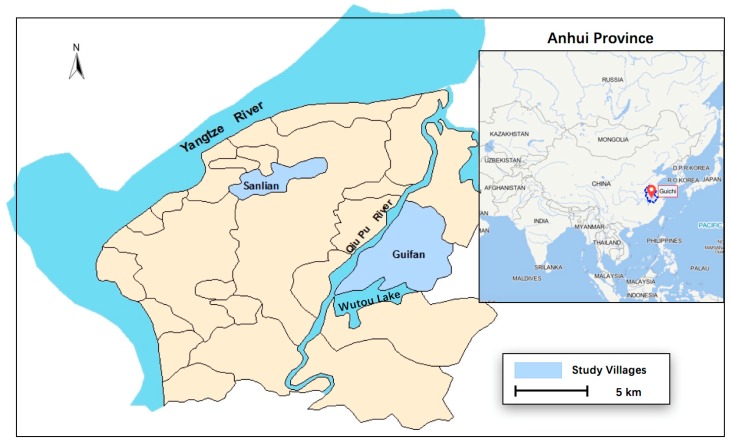
Location of the study area in Guichi district, Anhui Province, China.

The study was conducted in a marshland area including Sanlian village (30.642447, 117.346828 and Guifan village (30.574982, 117.410960), in Guichi district, Anhui Province, which is located in the middle and lower reaches of the Yangtze River Basin, the largest river in China (Figure 1). 

Its humid subtropical monsoon climate has annual average temperatures of 16 degrees Celsius and annual average rainfall of 1600 mm. This provides a suitable environment for the survival of Schistosoma japonicum. There are many small hills full of swamps, ponds and ditches in Guichi. This feature makes Guichi a typical schistosomiasis epidemic area. In addition, people in Guichi live along rivers or lakes, and their lifestyles area mainly involve farming and grazing, fishing and lake grassing. These labor activities expose local residents to be infected by schistosomiasis [7]. At the end of 2012, the total habitat area of snails in Guichi district was approximately 26.2715 km^2^ [8]. Of 650,000 people in Guichi, about 400,000 people were at risk of schistosomiasis, and the endemic area covered 16 towns and 165 villages [8]. All subjects gave their informed consent for inclusion before they participated in the study. The research was approved by the Medical Ethics Committee of Fudan University School of Public Health (IRB#2011-03-0270).

### 2.2. Intervention Measures

Sanlian village applied molluscicide (solid pentachlorophenol) on snails’ habitats every spring during 1981–1984, and simultaneously administered praziquantel to people with positive fecal examination. Guifan village only administered praziquantel to subjects with positive fecal tests. After 1984, the different control measures were all stopped, and two villages began to follow the same control strategies: (1) by local government in 1985–1991. (2) recommended by the World Bank Loan Project (WBLP) in 1992–2001. (3) by the continued WBLP measures in 2002–2004. (4) since 2005, the integrated schistosomiasis control strategy has been carried out. Table 1 provides detailed information on the intervention measures from 1981 to 2017. 

### 2.3. Disease Diagnosis

During the study period, after the transmission season (October–November), residents aged 5 to 65 years (743 in Sanlian, 1479 in Guifan in 1981) were tested by Kato-Katz (KK) fecal test and/or indirect hemagglutination assay (IHA) [9]. After 1981, the number of examined people was about 600 to 1200 in Sanlian and Guifan, respectively. From 1981 to 1994, all subjects in the two villages were tested by the two Kato-Katz thick smears test. During the period of 1995–2004, subjects were screened by indirect hemagglutination assay method. From 2005 to 2014, all subjects were tested by three Kato-Katz thick smears test. All the subjects were screened by indirect hemagglutination assay and the seropositive were confirmed by Kato-Katz three smears test from 2015 to 2017. 

### 2.4. Statistical Analysis

The prevalence of human infection was estimated by the formula: (1) Observed prevalence (%) = No. of egg-positive/No. of examined people × 100% (by using Kato-Katz method). (2) Observed prevalence (%) = (No. of blood-positive/No. of examined people × 100% (by using IHA test). (3) Observed prevalence (%) = (No. blood-positive/No. of examined people by using IHA test) × (No. of egg-positive/No. of examined people by using Kato-Katz method) × 100% (by using Kato-Katz and IHA).

The adjusted prevalence is the observed prevalence of human infection after taking the sensitivity and specificity of the Kato-Katz method into consideration. The adjusted formula: *p*’ = $\frac{P_{0}+\beta -1}{\alpha +\beta -1}$ *p*’: adjusted prevalence, *p_0_*: observed prevalence, α: sensitivity, β: specificity [10]. 

Two stool samples with three thick smears each are used as the diagnostic gold standard [11]. The sensitivities of two smears and three smears Kato-Katz methods are 64% and 75% separately. The specificities are 100% for both of them; the sensitivity and specificity of IHA and the observed prevalence measured by it are not satisfy the condition of adjusted formula [10], therefore, the results of IHA cannot be adjusted.

All data were entered and built a database with Microsoft Excel, and statistical analyses were performed with the use of SPSS (version25, IBM, New York, NY, USA) and SAS software (University Edition, The Statistical Analysis System, Raleigh, NC, USA). We used the chi-square test, including testing for trend when appropriate, to examine any difference and change over time. Two-sided *p* values were calculated for all comparisons [12,13,14].

## 3. Results

### Infection in Humans

**Figure 2:**
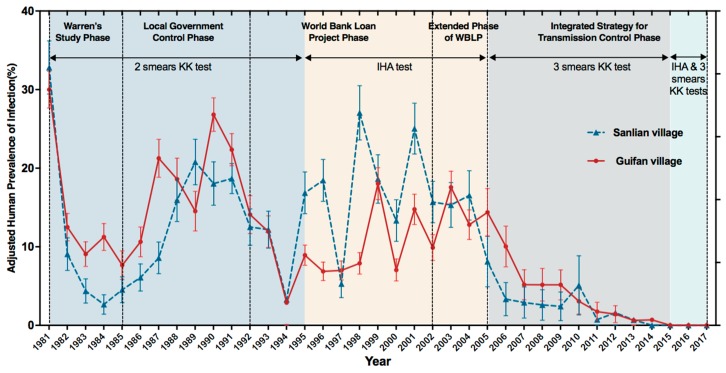
Prevalence of *Schistosoma japonicum* infection in humans in two villages during 1981–2017. (1) The adjusted prevalence formula: *p*’ = $\frac{P_{0}+\beta -1}{\alpha +\beta -1}$ *p*′: Adjusted prevalence; *p_0_*: Observed prevalence; α: Sensitivity; β: Specificity [10]; (2) The Kato-Katz (KK) 2 slides method was used in 1981–1994: α = 0.64, β = 1 [11]; The indirect hemagglutination assay (IHA) method was used in 1995–2004, but IHA not meet the condition of adjusted formula [10]; The Kato-Katz 3 slides method was used in 2005–2017, α = 0.75, β = 1 [11]; (3) Prevalence were assessed after the transmission season for each year in Sanlian and Guifan villages. (4) The I bars represent 95% confidence intervals.

The trend of schistosomiasis infection rates in the residents of Sanlian and Guifan villages over the last 37 years was shown in Figure 2. In 1981, the prevalence of schistosomiasis was 32.81% in Sanlian village and 30.00% in Guifan village (*χ*^2^ = 1.839, *p* = 0.175). With the intervention measures of human chemotherapy and snail control in Sanlian village, the prevalence dropped to 2.66% in 1984, while in Guifan village with chemotherapy only, the prevalence was only reduced to 11.25% (*χ*^2^ = 41.576, *p* = 0). By 1987, the prevalence in Guifan village increased to 21.25%, while the rate in Sanlian village rose to 20.78% until 1989. Since 1987, the local government in Guifan village has strengthened its control work so the rate declined again. Since the implementation of WBLP in 1992, the prevalence in Sanlian and Guifan villages had continued to decrease from 12.51% and 14.10% in 1992 to 3.29% and 2.89% in 1994. There was a volatile stage, with no obvious change trend in both villages from 1995 to 2004. In 1998 and 1999, infection rates in Sanlian and Guifan reached their respective peaks. A comprehensive control strategy has been implemented since 2005, and the prevalence decreased from 8.12% in 2005 to 0 in 2014 in Sanlian and from 14.39% to 0.70% for the same period in Guifan. There was no infected person detected in either village from 2015 to 2017. Both prevalences had significant downtrends since 2005 (Sanlian *χ*^2^ = 7.995, *p* = 0.005; Guifan *χ*^2^ = 9.433, *p* = 0.002).

## 4. Discussion

This study provides a long and continuous evaluation of the prevalence of schistosomiasis in China. Using disease data from 1981 to 2017, we found the changes of infection rates of humans within each historical phase were on account of different and complex causes, particularly social, economic, political, and health aspects. The schistosomiasis control strategies were suitable for the respective situations back then. Compared to most other studies concentrating on a single epidemic period or data from specific time points, infection rates and control strategies of schistosomiasis in this study are evaluated in a more comprehensive, systematic, and direct way. We confirmed that snail control and chemotherapy in combination has better effect than chemotherapy alone. In addition, we esteemed that the morbidity control measures taken in each historical period were appropriate and suited to the changing disease and social-economic status situations in China. We believed that WBLP is a successful example of disease control aided by foreign funds. Besides, we concluded the integrated strategy has greatly reduced the disease burden of schistosomiasis in China.

After different control measures were stopped in 1984, the prevalence in Sanlian village started to rise the next year, but still remained lower than that of the Guifan village until 1988. This suggests chemotherapy combined with snail control achieved robust and longer-term effects in reducing the prevalence. This conclusion corroborates control projects undertaken in other countries [15,16]. However, from 1987 to 1992, the prevalence oscillated in both villages, which was similar to other regions in China at that time. There are several possible reasons. First of all, since 1978, China’s rural areas have begun to shift from a centralized system to a contracted responsibility system for joint production. The mass extermination of snails is no longer practical. Moreover, as a result of responsibility privatization and the subsequent rise of labor cost, the previous schistosomiasis control measures become too expensive. Second of all, with the reform of China’s social and economic system in the 1980s, the source of schistosomiasis control budgets shifted from the previous three-level system (county, provincial, and the central governments) to the local government only, significantly intensifying the burden on local finances and therefore limiting corresponding expenditure on schistosomiasis control. Third of all, previous drug eradication of snails and environmental modification brought many problems such as environmental pollution and flooding, further increased economic burden and social impact [17]. Last but not least, praziquantel, a very effective chemotherapy drug started to be introduced to and widely used in China in the late 1980s [3], the Chinese government and the public dedicated less attention to schistosomiasis, which also partly led to the oscillation in prevalence. 

In 1992, large-scale drug treatment, health education and snails control were implemented in both villages, which led to obviously prevalence declined in the next two years. In 1990s, following the WHO’s recommendation that developing countries should utilize chemotherapy to achieve better morbidity control [18,19], a wide application of praziquantel, indirect hemagglutination, and the Kato-Katz method were initiated. This marked the starting point when China’s schistosomiasis control strategy switched from environmental modification to praziquantel-based control. Particularly, in 1992, with the help of the WHO, China began to implement the World Bank Loan Project (No. P003624) for “Infectious and Endemic Disease Control Project” [20,21], which provided 71 million dollars to help and support the government’s financial expenditure on schistosomiasis control, the application of parasite diagnostic techniques and the promotion of chemotherapeutic drugs [22]. Following a sharp decline in 1992–1994, the prevalence in the two villages rebounded in 1995 due to flooding in Guichi district. On the contrary, in 1997, the drought in Guichi decreased the risk of infection and therefore the prevalence. Similarly, prevalence peaked again in 1998 and 1999 as a result of severe flooding in China’s Yangtze River Basin that year [23]. Many new snail areas also appeared after the flood and this aggravated the disease burden [24]. As shown in Figure 2, prevalence in two villages oscillated at a high level from 1995 to 2004 even with continuous praziquantel-based control, indicating human chemotherapy alone might not be sufficient to completely eliminate schistosomiasis. This was also supported by another study [25]. Fluctuation of prevalence in the late WBLP period was also confirmed by another study [26]. There are four possible reasons for the changing prevalence at that time. Firstly, the reform of government institutions and the loss of well-trained technicians caused difficulties in normal operation of schistosomiasis control and budget reduction. Secondly, with the termination of the WBLP, the public’s compliance [27] with drug treatment had also decreased. Thirdly, although the prevalence and incidence of acute schistosomiasis dropped to a relatively low level in the early stage of this period [28], praziquantel could not stop transmission in high-epidemic areas and reinfection [3]. Lastly, natural factors such as floods also contributed heavily to the prevalence fluctuation. 

From 2005 to 2017, the infection rates in both villages decreased significantly. This might result from four aspects. First of all, in 2004, Chinese government set up four priority control programs of communicable diseases including schistosomiasis, AIDS, tuberculosis and hepatitis B [14], and schistosomiasis received an increased attention. Second of all, Chinese government launched the “National long-term plan for control of schistosomiasis (2004–2015)” program [29] in response to the rebounded trend of prevalence, which marked the beginning of the integrated control strategy in the country. Third of all, as the government increased its public health budget for schistosomiasis, the government transfer payment program was started and the national health system was strengthened. Lastly, the comprehensive control strategy, including praziquantel treatment of human and animals, molluscide application, health education, sanitation improvement, removal of bovines, etc., were implemented in our study area in 2006 [30] which greatly helped to reduce the disease burden of schistosomiasis. This result is consistent with the conclusions of many other studies [6,30,31,32,33,34,35,36,37,38]. However, several issues remain as well. Firstly, the comprehensive control strategy is not able to further reduce the infection rate to less than 3% in some areas [30,39]. Secondly, although removal of agriculture main livestock, such as bovines, sheep, and horses, can reduce human prevalence to a large extent [24], the existing control measures need to be adjusted in China since 45 species of mammal can be infected by *S*. *japonicum* in total, including but not limited to dogs, cats, and rodents [40,41,42]. Thirdly, the broadly used Kato-Katz method has relatively low sensitivity, particularly for subjects with low infectiosity [43,44]. This could lead to false negative results. Recently, several studies confirmed that Helmintex method is a more sensitive egg detection procedure than Kato-Katz method and other diagnostic tests like the point-of-care immunodiagnostic for detecting schistosome cathodic circulating antigen (POC-CCA) and Saline gradient [45,46]. Perhaps widely used of Helmintex in China is feasible and necessary. Fourthly, impact of water conservancy project on schistosomiasis control still remains controversial, due to many influencing factors such as water level, temperature, and plants change [47]. Lastly, environmental change, migration of population, and effectiveness of drug treatment could also influence the transmission of *S. japonicum*. Further investigations and effective long-term policies in controlling these factors are needed. 

The main limitation of our study is that the two villages are both typical bottomland areas and cannot represent all types of epidemic areas. In addition, a comparative study of the two villages was only run from 1981 to 1984. Therefore, there is no comparison group afterwards. Moreover, the study used three different diagnostic methods: two slides Kato-Katz, indirect hemagglutination assay (IHA), and three slides Kato-Katz, however, infection rate tested by IHA cannot be adjusted with its sensitivity and specificity [10]. 

## 5. Conclusions

Our results demonstrated that Guichi district has implemented suitable schistosomiasis control strategies for the respective social circumstances in different periods. This study suggested that adjusting the control strategy in according to social, economic, and political changes is necessary and vital. Schistosomiasis control in Guichi district is one of the successful examples in schistosomiasis control over the past 40 years in China. It could provide valuable experience on future policy making not only for China, but also epidemic areas with similar social conditions around the world. 

## Figures and Tables

**Table 1:** The varieties of control strategies of schistosomiasis from 1981–2017 in Guifan and Sanlian villages in China.

Time	Phase	Specific Methods
1981–1984	Warren’s Study Control	Guifan Village: People with a positive stool examination result should receive drug chemotherapy. Sanlian Village: 1. People with positive result of stool examination should receive drug chemotherapy. 2. The areas where snail lived were sprayed with molluscicide every spring.
1985–1991	Local Government Control	1. Chemotherapy for people and cattle was the basic measure. 2. Health education for people. 3. Molluscicide was directed at the susceptible areas.
1992–2001	World Bank Loan Project	Goal: Enhanced morbidity control through praziquantel to human and bovines. Details: 1. Chemotherapy was complemented by health education. 2. Snails were controlled by environmental management. 3. Molluscicide was a key way to sustain transmission control.
2002–2004	Extended Period of WBLP Control	1. Achievements from WBLP were not reinforced. 2. The extent of chemotherapy shrinked and compliance of chemotherapy became worse. 3. Returning farmland to lake was advocated due to the molluscicide and previously reclaimed land from the lake.
2005–2017	Integrated Strategy Control	2005: 1. Chemotherapy with praziquantel for people and cattle. 2. Snail control with molluscicides. 3. Health education programmes—residents should stay away from snail-infested areas and water Since 2006: 4. All cattle were replaced with mechanized equipment. 5. Other domestic animals were fenced in. 6. Piped water and lavatories were supplied to improve sanitation.
